# Identification of a Second PIP Motif Reveals New Insights into PCNA Recognition by Yeast CAF-1

**DOI:** 10.3390/biom16091274

**Published:** 2026-09-03

**Authors:** Ian Hall, Iain M. Davies, Stephanie A. Limaye, Ivy L. Williams, Cael E. Carlson, Trevor J. Snetsinger, Andy E. Agbakpo, James W. Checco, Lynne M. Dieckman

**Affiliations:** 1 Department of Chemistry and Biochemistry, Creighton University, Omaha, NE 68178, USA; 2 Department of Chemistry, University of Nebraska-Lincoln, Lincoln, NE 68588, USA; aagbakpo2@huskers.unl.edu (A.E.A.); checco@unl.edu (J.W.C.); 3 Nebraska Center for Integrated Biomolecular Communication, University of Nebraska-Lincoln, Lincoln, NE 68588, USA

**Keywords:** chromatin assembly factor 1 (CAF-1), proliferating cell nuclear antigen (PCNA), nucleosome assembly, PCNA-interacting peptide (PIP)

## Abstract

Proliferating cell nuclear antigen (PCNA) is an essential sliding clamp that coordinates nearly all DNA-templated processes. It does so by recruiting a diverse array of factors to DNA through PCNA-interacting protein (PIP) motifs on PCNA-binding proteins. Chromatin assembly factor 1 (CAF-1) is a histone chaperone that deposits histones onto silent regions of the genome immediately following DNA replication. PCNA recruitment of CAF-1 to the replication fork is essential for nucleosome assembly and epigenetic inheritance. In yeast, CAF-1 recruitment to PCNA is assumed to be facilitated using one PIP motif. Here, we identify a second PIP motif in CAF-1, designated PIP1, located within the N-terminal intrinsically disordered region of the protein. Binding kinetics demonstrate that the PIP1 motif sequence binds PCNA with substantially lower affinity than the previously characterized PIP motif, designated PIP2. Structural and binding data reveal PIP1 as an extended PIP motif, in which C-terminal flanking residues make extensive, atypical contacts with PCNA that significantly increase its affinity. Neither PIP1 nor PIP2 alone is required for CAF-1–mediated gene silencing in vivo, but simultaneous disruption of both abolishes CAF-1 function. Together, these findings suggest CAF-1 engages PCNA multivalently and provide new insight into how PCNA selectively recognizes binding partners during nucleosome assembly.

## 1. Introduction

Proliferating cell nuclear antigen (PCNA) is a key modulator of DNA metabolic processes [1]. It acts as a sliding clamp that encircles DNA and tethers numerous proteins to replication forks. The structure of PCNA is homotrimeric, where each subunit of PCNA contains two similar domains that are linked to each other by an interdomain connecting loop (IDCL) [2]. In addition to its canonical role in enhancing DNA polymerase processivity, PCNA serves as a molecular scaffold that recruits and regulates the function of a diverse array of factors involved in DNA replication, repair, and chromatin assembly [3]. This recruitment is largely mediated through short interaction motifs on PCNA-binding proteins called PCNA-interacting protein (PIP) motifs. A PIP motif is a conserved sequence of eight amino acids, typically represented by the consensus sequence Qxx[L/I/M]xx[F/Y][F/Y], where “x” represents any amino acid. A majority of PCNA-binding motifs in PCNA-binding proteins are located within intrinsically disordered regions (IDRs) or are flanked by IDRs [4].

**Figure 1:**
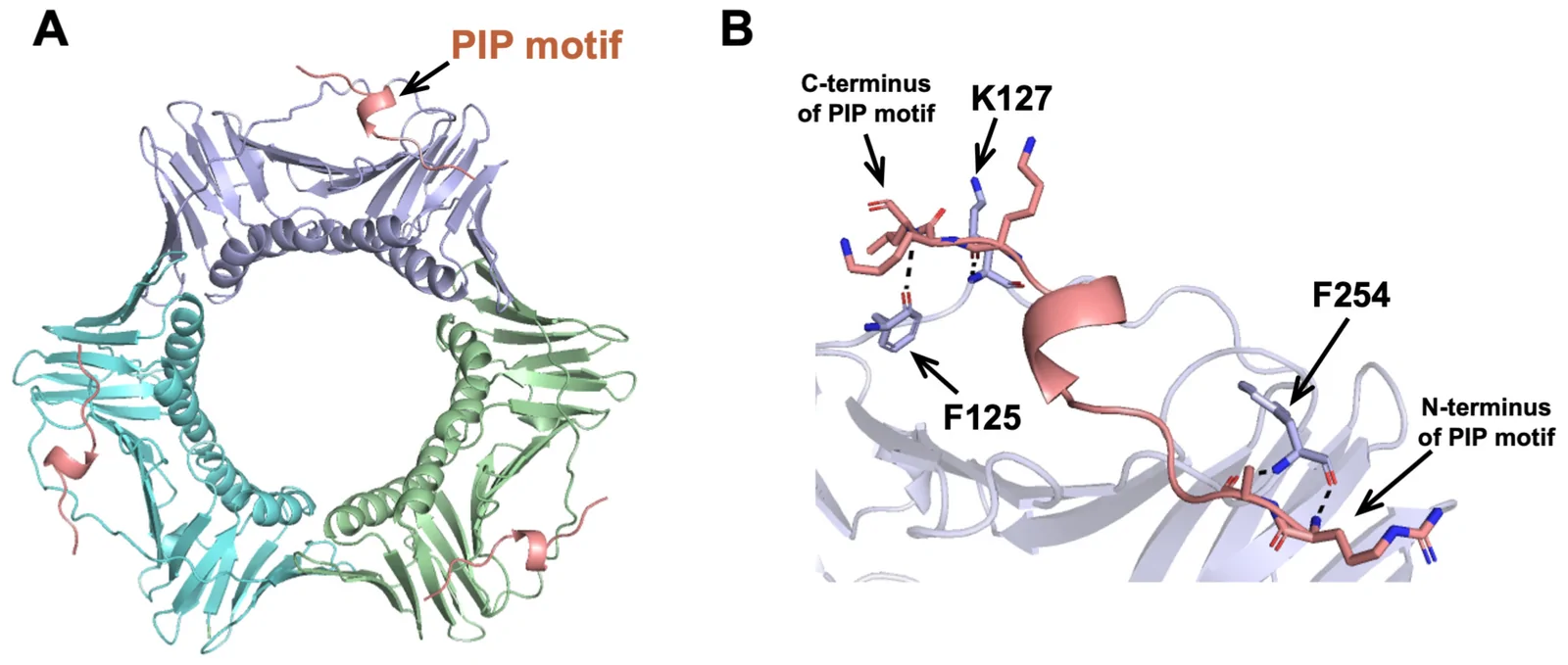
Structure of the known PIP motif of CAF-1 bound to PCNA (PDB 8THW). (**A**) Three PIP motifs (pink) bound to one PCNA trimer. Each subunit of PCNA is represented by a different color (purple, teal, light green). (**B**) Close-up of one PIP motif of CAF-1 (pink) bound to PCNA (purple). Polar interactions (black dashed lines) between residues N-terminal to the PIP motif bound to the C-terminus (residue F254) of PCNA and residues C-terminal to the PIP motif bound to the IDCL (residues F125 and K127) of PCNA. Resource: https://www.rcsb.org/structure/8THW (accessed on 10 June 2026).

Canonical PIP motifs bind a hydrophobic pocket on the front face of PCNA near the IDCL through the conserved glutamine, aliphatic, and aromatic residues in positions one, four, seven, and eight. Upon binding PCNA, these motifs form a characteristic 3_10_ helix (Figure 1A). Despite the short length and the high degree of sequence conservation among PIP motifs, PCNA is able to reliably discriminate between these motifs and coordinate an extraordinary number of factors that compete for access to DNA. This raises fundamental questions about how PCNA can achieve selectivity of binding partners at the replication fork during DNA-templated processes. Accumulating evidence indicates that variability among PIP–PCNA interactions may be due to differences in amino acids immediately flanking the PIP motifs [4,5,6,7].

Chromatin assembly factor 1 (CAF-1) is a key PCNA-binding protein that directly links DNA synthesis to chromatin assembly. CAF-1 is a heterotrimeric histone chaperone complex responsible for depositing newly synthesized, modified histones H3 and H4 onto nascent DNA immediately following replication, thereby maintaining nucleosome organization and preserving epigenetic information. The interaction between CAF-1 and PCNA is essential for replication-coupled nucleosome assembly and heterochromatin maintenance [8,9]. Disruption of this interaction impairs chromatin formation and gene silencing [8,10,11]. In yeast, CAF-1 is composed of subunits Cac1, Cac2, and Cac3. CAF-1 recruitment to PCNA at replication forks is believed to be mediated through a PIP motif located near the middle of its largest subunit, Cac1 [12,13]. We solved the structure of this motif bound to PCNA previously (Figure 1A) [11].

Previous studies of PIP–PCNA interactions have demonstrated that residues immediately flanking PIP motifs are important in modulating PCNA interactions [4,5,6,7]. These sequences may influence both affinity and specificity by binding secondary surfaces on PCNA, such as the C-terminus or IDCL [14,15]. In the case of CAF-1, residues on the N-terminal and C-terminal side of the PIP motif in Cac1 make contacts with the C-terminus and the IDCL of PCNA, respectively (Figure 1B) [11]. Furthermore, positively charged residues adjacent to the PIP motif appear to enhance PCNA binding, whereas negatively charged residues can inhibit a more robust interaction [11].

In humans, CAF-1 consists of the three subunits p150, p60, and p48. The largest subunit, p150, contains two PIP motifs, denoted PIP1 and PIP2 [13,16]. Previous studies have shown these two PIP motifs are distinct, where the canonical PIP2 motif exhibits low affinity binding to PCNA and is critical for replication-coupled chromatin assembly, while the non-canonical PIP1 motif exhibits higher affinity to PCNA and may be dispensable for CAF-1 recruitment and gene silencing [16]. The significance of possessing two PIP motifs with differing affinities and functions is not clear.

Here, we identified a second PIP motif in yeast Cac1. This motif is located within the N-terminal, intrinsically disordered region of the protein. Therefore, we denote the motif as PIP1. Biochemical analyses show that the PIP1 motif binds PCNA with substantially lower affinity than the previously characterized PIP2 motif. However, data suggest that the PIP1 motif adopts an extended conformation, possibly forming a β-sheet interaction with the IDCL of PCNA that increases the affinity of the interaction and distinguishing it from most other known PIP motif–PCNA interactions. Binding studies show that the presence of the extended residues increases the affinity of the PIP1 motif for PCNA by approximately four-fold. Functional assays indicate neither the extended PIP1 motif nor the PIP2 motif alone is required for CAF-1–mediated gene silencing in vivo; however, CAF-1 is not functional when both motifs are disrupted. Together, these studies suggest a multivalent role for the PIP motifs in CAF-1 and enhance our understanding of how PCNA recognizes its binding partners at the replication fork.

## 2. Materials and Methods

### 2.1. Protein Expression and Purification

Wildtype yeast PCNA was N-terminally His_12_-tagged and overexpressed in BL21 (DE3) bacteria harboring plasmid pKW336. Transformed cells were induced by the addition of IPTG at 37 °C for 3 h (LB media). Cells were lysed via sonication, and the cell lysate was subjected to centrifugation for clarification. PCNA was purified using Ni-NTA agarose affinity chromatography (Thermo Scientific, Waltham, MA USA), DEAE sepharose fast flow anion exchange chromatography (GE Healthcare, Uppsala, Sweden), and a Superdex 200 size exclusion chromatography column (GE Healthcare). To produce the PCNA–PIP1 fusion proteins, a five amino acid linker (GGSGG) and the sequence of the extended PIP1 motif of yeast CAF-1 were cloned, respectively, in frame, at the C-terminus of His_6_-tagged PCNA in the plasmid pKW336. The fusion protein was overexpressed in BL21 (DE3) cells. Cells were lysed via sonication, and the cell lysate was subjected to centrifugation for clarification. The PCNA–PIP fusion protein was purified using Ni-NTA agarose affinity chromatography (Thermo Scientific), DEAE anion exchange chromatography (GE Healthcare), and a Superdex 200 size exclusion chromatography column (GE Healthcare).

### 2.2. Peptide Synthesis

The CAF-1 PIP1 peptides used in the initial surface plasmon resonance (SPR) experiments (residues 12–26, Figure 3) and the PIP1β peptides used for Figure 7C–E were commercially synthesized by GenScript USA Inc. (Piscataway, NJ, USA). All other CAF-1 PIP1 peptides were manually synthesized by Fmoc solid-phase peptide synthesis on Rink Amide Protide resin (CEM Corporation, Matthews, NC, USA). Before synthesis, the resin was swelled in N, N-dimethylformamide (DMF) for 30 min. Each coupling reaction utilized four equivalents of Fmoc-protected amino acid from Aapptec, Novabiochem, Chem-Impex, or Anaspec, four equivalents of HCTU (O-(1H-6-chlorobenzotriazole-1-yl)-1,1,3,3- tetramethyluronium hexafluorophosphate), and eight equivalents of N, N-diisopropylethylamine in 4 mL of DMF. Coupling reactions were conducted at room temperature for 40 min to 24 h with constant stirring, followed by rinsing the resin with 4 mL of DMF three times. Fmoc deprotection reactions were carried out using 20% piperidine in DMF for 20 min at room temperature with constant stirring, followed by rinsing the resin with 4 mL of DMF three times. After coupling the last amino acid, peptides were cleaved from the resin using 95% trifluoroacetic acid (TFA), 2.5% triisopropylsilane, and 2.5% water (H_2_O) for a duration of 4–6 h with constant stirring. Cleaved peptides were precipitated with ice-cold diethyl ether followed by centrifugation at 3000× *g* for 10 min at 4 °C. The diethyl ether was then removed by decantation, followed by air drying to obtain a pellet. The peptide pellet was then dissolved in 50% acetonitrile (ACN)/H_2_O mixture and filtered using a 0.22 µm syringe filter from Fisher Scientific. After filtration, the peptides were purified by reverse-phase high-performance liquid chromatography (HPLC) using a 1260 Infinity II Preparative LC System from Agilent Technologies (Memphis, TN, USA) fitted with a Pursuit 5 µm, 200 Å, 250 × 21.2 mm C18 column from Phenomenex. The final purity of the peptides was determined by analytical-scale HPLC using a 1260 Infinity II LC System from Agilent Technologies fitted with a Pursuit 3 200 Å, 150 × 4.6 mm C18 column from Phenomenex (Torrance, CA, USA). The mobile phase for both types of HPLC runs consisted of Solvent A (H_2_O + 0.1% TFA) and Solvent B (ACN + 0.1% TFA). HPLC runs were accomplished by gradient elution with an increasing concentration of Solvent B. For analytical HPLC runs, the concentration of Solvent B was increased in a linear gradient from 5 to 55% over the course of 30 min or 60 min at a constant flow rate of 1 mL/min. The identities of the synthesized peptides were confirmed via matrix-assisted laser desorption ionization mass spectrometry using a Bruker solariX XR Fourier transform ion cyclotron resonance mass spectrometer (Bruker Corporation, Billerica, MA, USA) (see Supplemental Figure S1). Peptides were quantified through UV-Visible spectroscopy using a VWR UV-3100PC spectrophotometer (VWR International LLC, Radnor, PA, USA). For quantification, a tryptophan residue was added to WT PIP1 (10–27), WT PIP1 (10–32), and WT PIP1 (10–40). These were dissolved in water and quantified using ε_280nm_ = 5690 M^−1^cm^−1^ [17]. The peptide used in Figure 7B (*β; residues 10–40) lacked a tryptophan residue. This was dissolved in water and quantified using an estimated ε_214nm_ based on the contributions from all peptide bond linkages [18,19].

### 2.3. Surface Plasmon Resonance

SPR assays were performed using a Biacore 8K (Cytiva, Uppsala, Sweden). Experiments were carried out at a flow rate of 30 μL/min at 25 °C in 10 mM HEPES, pH 7.4, 300 mM NaCl, 50 μM EDTA, 0.05% surfactant P20, and 1 mM DTT as the running buffer. His_12_-tagged PCNA was immobilized on an NiHC200M sensor chip (XanTec bioanalytics, Düsseldorf, Germany). The chip surface was first activated using an injection of 350 mM EDTA (25 µL/min), followed by an injection of 0.5 mM NiCl_2_ (15 µL/min). Increasing concentrations of the PIP1 peptide in running buffer were injected over the immobilized PCNA. Contact time for the PIP1 peptide was set to 60 s, and dissociation time was 180 s. The signal from buffer alone (containing no peptide) over a reference flow cell was subtracted from all responses. Kinetic binding constants were determined using non-linear least-squares fitting to a 1:1 binding model with the Biacore Insight Evaluation 5.0 software program. Steady state binding constants were obtained by fitting equilibrium data to a non-linear regression with GraphPad Prism 11.0.2. Experiments were performed using a minimum of seven replicates.

### 2.4. Small-Angle X-Ray Scattering

Wildtype and mutant fusion proteins used in SAXS contained residues 12–37 of CAF-1 fused to the C-terminus of a PCNA monomer via a five amino acid linker, GGSGG. SAXS was performed at BioCAT (beamline 18ID at the Advanced Photon Source, Chicago, IL, USA) with in-line size exclusion chromatography (SEC) to separate sample from aggregates and other contaminants, thus ensuring optimal sample quality and multiangle light scattering (MALS), dynamic light scattering (DLS), and refractive index measurement (RI)) for additional biophysical characterization (SEC-MALS-SAXS). The samples were loaded on a Superdex 200 Increase 10/300 GL column (Cytiva) run by a 1260 Infinity II HPLC (Agilent Technologies) at 0.6 mL/min. The flow passed through (in order) a MALS detector and a DLS detector (DAWN Helios II, Wyatt Technologies, Goleta, CA, USA), an RI detector (Optilab T-rEX, Wyatt), and a fiber-coupled UV cell. The flow then went through the SAXS flow cell. The flow cell consists of a 1.0 mmID quartz capillary with ~20 μm walls. A coflowing buffer sheath is used to separate the sample from the capillary walls, helping prevent radiation damage [20]. Scattering intensity was recorded using a PILATUS3 X 1M (Dectris, Baden-Daettwil, Switzerland) detector, which was placed 3.6 m from the sample, giving us access to a q-range of 0.003 Å^−1^ to 0.42 Å^−1^ at an energy of 12 keV. Then, 0.5 s exposures were acquired every 1 s during elution, and data were reduced using BioXTAS RAW 2.3.0 [21]. Buffer blanks were created by averaging regions flanking the elution peak and subtracted from exposures selected from the elution peak to create the I(q) vs. q curves used for subsequent analyses. REGALS was used to properly subtract buffer regions from samples with sloping baselines. Molecular weights and hydrodynamic radii were calculated from the MALS and DLS data, respectively, using the ASTRA 8 software program (Wyatt).

### 2.5. Nano Differential Scanning Fluorimetry

Nano differential scanning fluorimetry (nanoDSF) was performed using a Prometheus Panta instrument (NanoTemper Technologies, Munich, Germany). PCNA was buffer-exchanged into 50 mM TrisCl, pH 8.0, and 150 mM NaCl and diluted to varying concentrations ranging from 15 to 30 µM. The PIP1 motif peptide was dissolved in the same buffer and added in molar excess of at least 3.33-fold relative to PCNA. PCNA and peptide were mixed and incubated at 25 °C for 10 min prior to loading into standard nanoDSF-grade capillaries (NanoTemper Technologies). Samples were subjected to a thermal ramp from 25 °C to 95 °C at a rate of 1 °C/min. Intrinsic tryptophan fluorescence was monitored at 330 nm and 350 nm, and the ratio of fluorescence intensities (F350/F330) was recorded as a function of temperature. Melting temperatures (T_m_) were determined from the first derivative of the F350/F330 ratio using the Prometheus Panta analysis program (PR.Panta Analysis 1.10.3). Shifts in T_m_ (ΔT_m_) relative to PCNA alone were used as an indicator of binding, with a positive ΔT_m_ value indicating stabilization of PCNA upon peptide binding. Experiments were performed with n = 4 independent experiments, and mean ΔT_m_ values are reported with standard deviation.

### 2.6. Gene Silencing Assays Using Flow Cytometry

A yeast strain containing a deletion of the endogenous *cac1* gene (W303, *cac1D::leu2*, *rtt106::kan*, *hmr::GFP/URA3*) and a pRS313-derived yeast expression plasmid for the wildtype Cac1 protein were kindly provided by Bruce Stillman. A Q5 Site-Directed Mutagenesis Kit (NEB, Ipswich, MA, USA) was used with the yeast expression plasmid as a template to generate a plasmid with the ΔPIP2 motif mutation in the *cac1* gene. All other plasmids encoding mutated Cac1 proteins were purchased from GenScript. The *cac1Δ* yeast cells were then transformed with either the wildtype plasmid or one of the mutant Cac1 plasmids using a Frozen-EZ Yeast Transformation kit (Zymo Research, Irvine, CA, USA). For controls, yeast cells were left untransformed or transformed with the pRS313 empty vector. Cells were grown in 3 mL of liquid yeast peptone dextrose (YPD) media overnight with incubation at 30 °C and shaking at 200 rpm. Overnight cultures were used to inoculate fresh 10 mL cultures to OD_600_ ≈ 0.2 and were grown under the same incubation conditions to OD_600_ ≈ 0.7 (early log phase). Cells were pelleted by centrifugation at 4500 rpm for 5 min at 4 °C. The cell pellets were washed 3× with 2 mL of 1× PBS (137 mM NaCl, 2.7 mM KCl, 10 mM Na_2_HPO_4_, 1.8 mM KH_2_PO_4_ pH = 7.4) by resuspension and pelleting using the centrifugation conditions described above. Intercellular aggregates were diminished by water bath sonication (Bransonic CPX2800H, Danbury, CT, USA) for 5 min at 25 °C on the low power setting. Next, 20–50 μL of sonicated yeast cells was added to 1 mL 1× PBS and 1 μL of propidium iodide (PI) (Invitrogen, Waltham, MA, USA) and incubated at 25 °C in the dark for 15 min. The cells were pelleted by centrifugation at 1200 rpm for 5 min, and excess PI in the supernatant was decanted. An additional 1 mL of fresh 1× PBS was used to resuspend the PI-treated yeast. For each culture, 200 uL of cell suspension was acquired using a ZE5 flow cytometer (Bio-Rad Laboratories, Hercules, CA, USA) enabled with a Forward Scatter Small Particle Detector (FSc spd). Data analysis of generated Flow Cytometry Standard (FCS) files was performed using Flowjo Version 10. For this, sequential gating was carried out to first exclude doublets. Next, dead cells were removed through exclusion of PI-positive cells. Finally, estimations of the percent frequency of GFP-expressing yeast were carried out. Experiments were performed a minimum of three times using independent yeast preparations.

## 3. Results

### 3.1. Identification of a Second PIP Motif in Cac1

**Figure 2:**
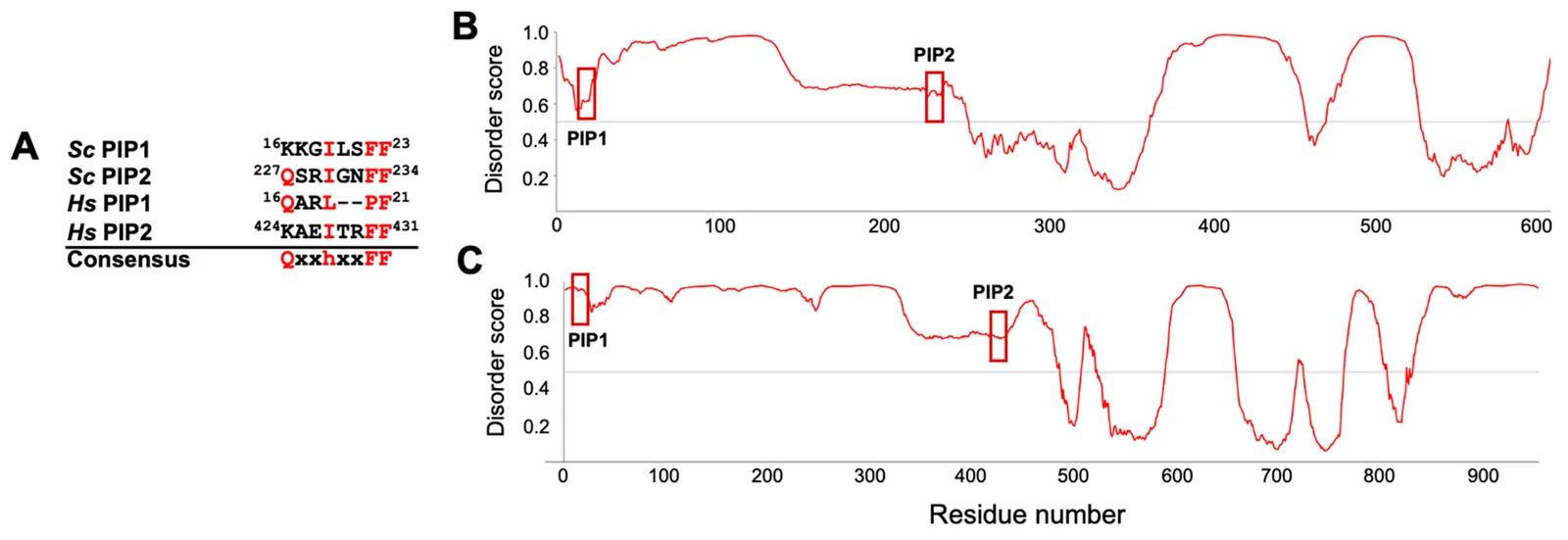
Locations and sequences of PIP motifs in yeast and human Cac1. (**A**) Sequences of the PIP1 and PIP2 motifs in yeast (Sc) and humans (Hs), with the consensus sequence of PIP motifs shown. Conserved residues are shown in red. Dashes indicate gaps in sequence, and an “x” indicates any amino acid. The disorder probability prediction plot was obtained using the AIUPred prediction tool [25,26] for the Cac1 subunits of CAF-1 in yeast (**B**) or humans (**C**). Locations of the PIP1 and PIP2 motifs in each protein are indicated. Regions with values below 0.5 (indicated by the horizontal line) are predicted to be structured, and regions with values above 0.5 are predicted to be disordered.

A small number of PCNA-binding proteins are known to contain more than one PIP motif, including human and yeast DNA polymerases and human CAF-1 [16,22,23,24]. We sought to determine whether yeast CAF-1 also contained more than one PIP motif. Using sequence analysis, we identified a second, putative PIP motif near the N-terminus of the Cac1 subunit with the sequence KKGILSFF (Figure 2A). Because this motif is N-terminal to the known PIP motif in Cac1, we named the N-terminal motif the “PIP1 motif” and the previously determined motif the “PIP2 motif.” Similarly, the PIP1 and PIP2 motifs of human CAF-1 exist near the N-terminus and near the center of the largest subunit, p150. As observed for most PIP motifs, both motifs in the yeast and human CAF-1 protein exist within predicted intrinsically unstructured regions (Figure 2B,C).

### 3.2. The PIP1 Motif of Cac1 Binds PCNA with Low Affinity

**Figure 3:**
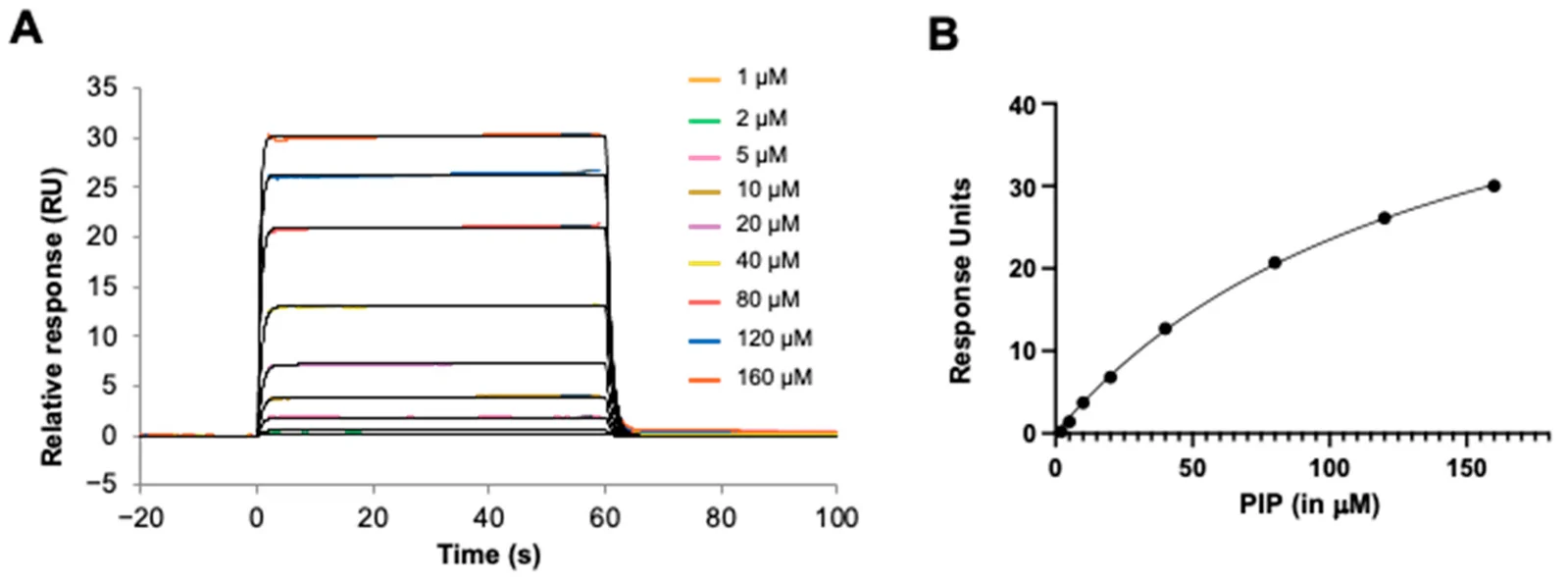
SPR binding studies of the CAF-1 PIP1 motif and PCNA. (**A**) SPR of the isolated CAF-1 PIP1 peptide (residues 12–26; DDTKKKGILSFFQNT) binding to immobilized PCNA. Increasing concentrations of the PIP1 peptide were injected onto PCNA captured on the chip. The signal from injection of buffer only (no PIP1 peptide) was subtracted from all data. Resulting curves were fit to a one-step binding equation (BIAevaluation 5.0 software; black lines). (**B**) Responses at equilibrium for each concentration of PIP1 peptide were plotted and fit to a non-linear regression to obtain the steady state affinity of the interaction.

Binding between the PIP1 motif of yeast CAF-1 and PCNA was measured quantitatively using SPR. PCNA was captured on the surface of a sensor chip, and increasing concentrations of a peptide containing the PIP1 motif (residues 12–26) were added to the chip (Figure 3A). The binding affinity of the PCNA–CAF-1 interaction, obtained by plotting the steady state response units vs. ligand concentration, was measured as K_d_ = 120 ± 10 μM (Figure 3B). This affinity was surprising, as this is significantly lower than all other known PIP–PCNA interactions. Binding affinities between other PIP motifs and PCNA have been determined to be as high as 80 nM or as low as 60 μM, with most interactions in the mid-micromolar range (1–60 μM) [4,27]. For comparison, the PIP2 motif of yeast CAF-1 binds PCNA approximately 30-fold tighter than the PIP1 motif, with an affinity of 4 μM [11].

Evaluation of the kinetics of the sensorgrams fit best to a 1:1 binding model. The association and dissociation rate constants were outside the lower and upper detection limits of the instrument, respectively, for a majority of the experiments. We did obtain reliable kinetic constants from one experiment (Table 1). The quality of the fit was confirmed by a low χ^2^ value of 0.01 (which was 0.02% of the Rmax), supporting the validity of the model, suggesting these values may be close to the true parameters. Importantly, the K_d_ obtained from kinetic rate constants (120 µM) was identical to the steady state K_d_ (120 µM) determined across multiple replicates, providing confidence in the overall affinity measurement independent of the kinetic analysis. Overall, these results are consistent with a weak, transient interaction typical of PIP–PCNA binding.

These kinetic analyses differ drastically from the PIP2 motif of CAF-1 binding to PCNA, which exhibited a two-step binding mechanism (Table 1). This suggests that association of the PIP2 motif to PCNA begins with a step that is relatively fast, followed by a much slower step involving a conformational change [11]. In contrast, the PIP1 motif binds PCNA using a one-step mechanism, without an additional conformational step.

### 3.3. Identification of a Putative, Extended PIP1 Motif

In an effort to determine the structural basis of the PIP1 motif of CAF-1 binding to PCNA, we initially pursued X-ray crystallography. This approach has been used successfully to characterize numerous PIP–PCNA complexes, including the CAF-1 PIP2 motif bound to PCNA. However, these efforts were unsuccessful, likely owing to the relatively low affinity and instability of the PIP1–PCNA complex. We therefore employed small-angle X-ray scattering (SAXS) to characterize the solution conformation of the PIP1–PCNA complex at low resolution.

**Figure 4:**
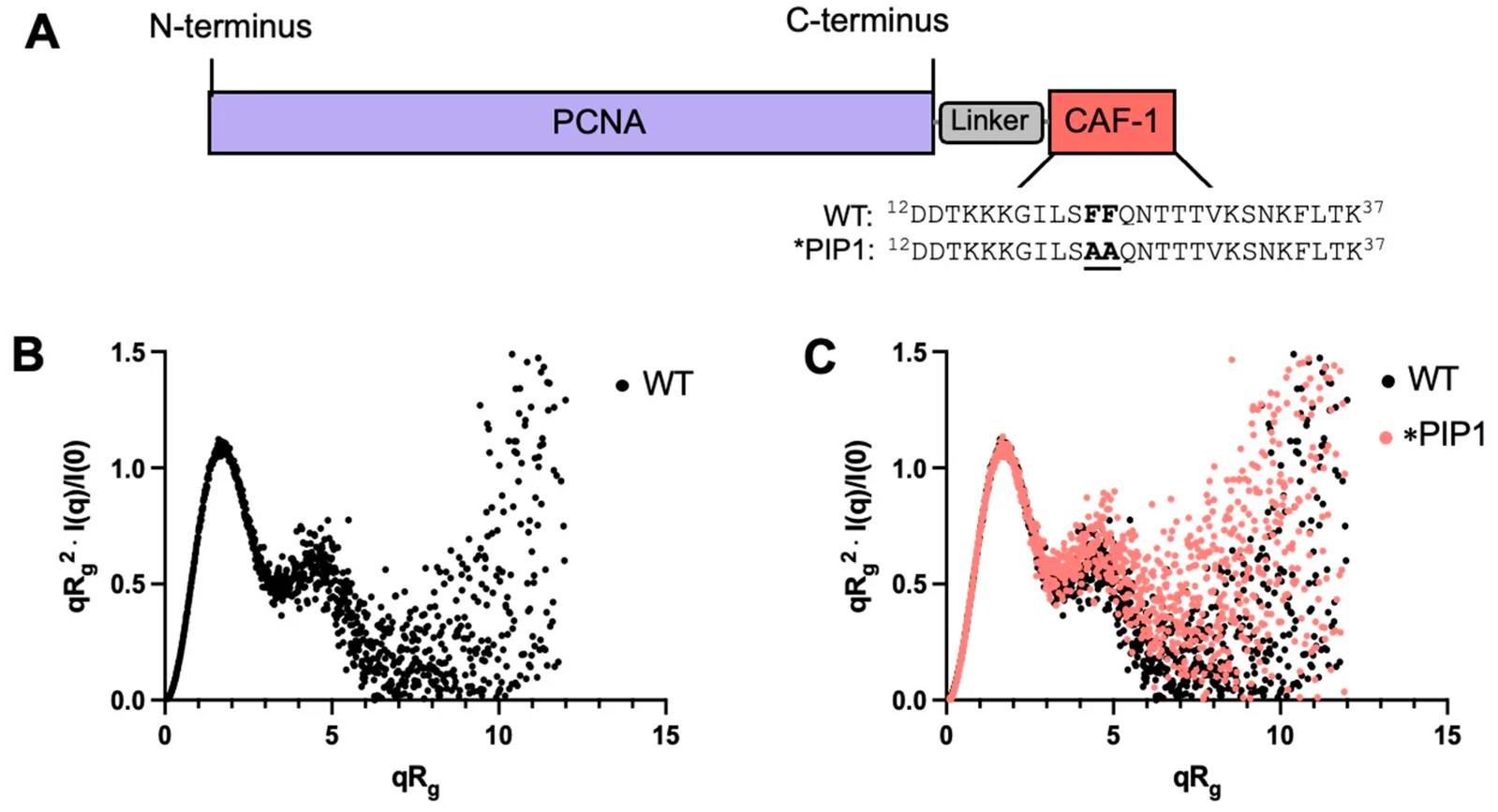
SAXS analysis of the PIP1 motif of CAF-1 bound to PCNA. (**A**) Linear representation of the PCNA–CAF-1 fusion proteins generated for SAXS experiments. A sequence containing the PIP1 motif of CAF-1 is shown in red, the five amino acid linker is shown in grey, and the PCNA monomer is shown in light purple. The wildtype and PIP1 mutant sequences are indicated, with the mutated residues bolded and underlined. (**B**) Dimensionless Kratky plot for the wildtype PCNA–CAF-1 fusion protein. (**C**) Kratky plot for the mutant PCNA–CAF-1 fusion protein overlaid with the wildtype protein.

One PCNA trimer contains three identical binding sites for PIP motifs. In addition, the C-terminus of PCNA is in very close proximity to the PIP-binding region in each subunit (see Figure 1B). Therefore, to ensure a stoichiometric ratio of three PIP motifs to one PCNA trimer in these complexes, we employed a method we previously developed for PIP–PCNA structural determination [11]. Here, a peptide sequence containing the PIP1 motif of CAF-1 was fused to the C-terminus of a PCNA monomer via a flexible linker (Figure 4A). To compare conformational changes between the PIP-bound and unbound states of PCNA, we also generated a mutant form of the PCNA–PIP1 fusion protein where the PIP1 motif was unable to bind PCNA. For this, the two phenylalanines in positions seven and eight in the PIP1 motif were mutated to alanine residues (*PIP1, Figure 4A). Previous studies have established that the double phenylalanine to alanine mutation in canonical PIP motifs inhibits binding to PCNA [11,28,29,30]. Figure 4B,C show dimensionless Kratky analysis of the wildtype and mutant PIP–PCNA complexes. Unexpectedly, the mutant protein complex exhibited the same overall conformation as the PIP-bound PCNA, as observed in Figure 4C, where overlay of the wildtype and PIP1 mutant SAXS data show no significant differences. These results suggest that an interaction may still be present in the complex, and that residues within the CAF-1 peptide but outside the PIP1 motif are interacting with PCNA.

**Figure 5:**
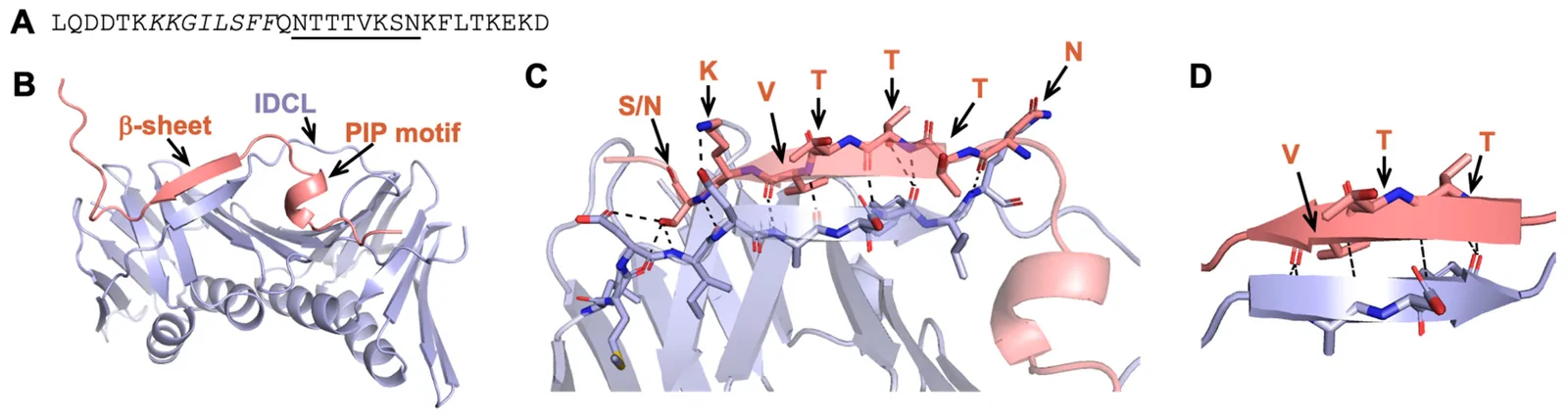
Predicted structure of the extended PIP1 motif of CAF-1 bound to PCNA by AlphaFold. (**A**) Sequence of the Cac1 peptide used for prediction, where the PIP1 motif is italicized, and the additional residues predicted to interact with PCNA are underlined. (**B**) Structure of the extended PIP1 motif (pink) bound to PCNA (purple), as predicted by AlphaFold3. The IDCL of PCNA and the β-sheet formed between the additional residues of the PIP1 motif and PCNA are indicated. (**C**) Predicted polar contacts between the residues C-terminal to the PIP1 motif and PCNA, indicated as dashed lines. (**D**) Close-up view of the β-sheet formed between the PIP1 motif and PCNA. Predicted polar contacts between the “TTV” residues of Cac1 and the IDCL residues of PCNA are shown as dashed lines.

We used AlphaFold3 to identify possible interactions between PCNA and CAF-1 residues adjacent to the canonical PIP1 residues. Interestingly, AlphaFold predicted that a region immediately C-terminal to the PIP1 motif forms an anti-parallel β-sheet with the IDCL of PCNA. As many as eight additional amino acids in CAF-1 are predicted to make polar contacts with the IDCL of PCNA (Figure 5). Therefore, based on both experimental and predicted results, we hypothesized that the PIP1 motif of CAF-1 is an extended PIP motif, and requires amino acids C-terminal to the motif itself to bind PCNA with higher affinity.

### 3.4. Affinity Between the Extended PIP1 Motif and PCNA

To determine whether residues C-terminal to the PIP1 motif of CAF-1 interact with PCNA and to measure the affinity of the extended PIP1 motif–PCNA complex, we carried out SPR experiments with PCNA and various length peptides containing the PIP1 motif of CAF-1 (residues 10–27, residues 10–32, and residues 10–40). To ensure that the presence of a negative charge at the C-terminus of each peptide did not interfere with the interaction with PCNA, each peptide was synthesized with an amide group at the C-terminus, which more closely mimics the peptide bond that would be present in the context of the full-length protein.

**Figure 6:**
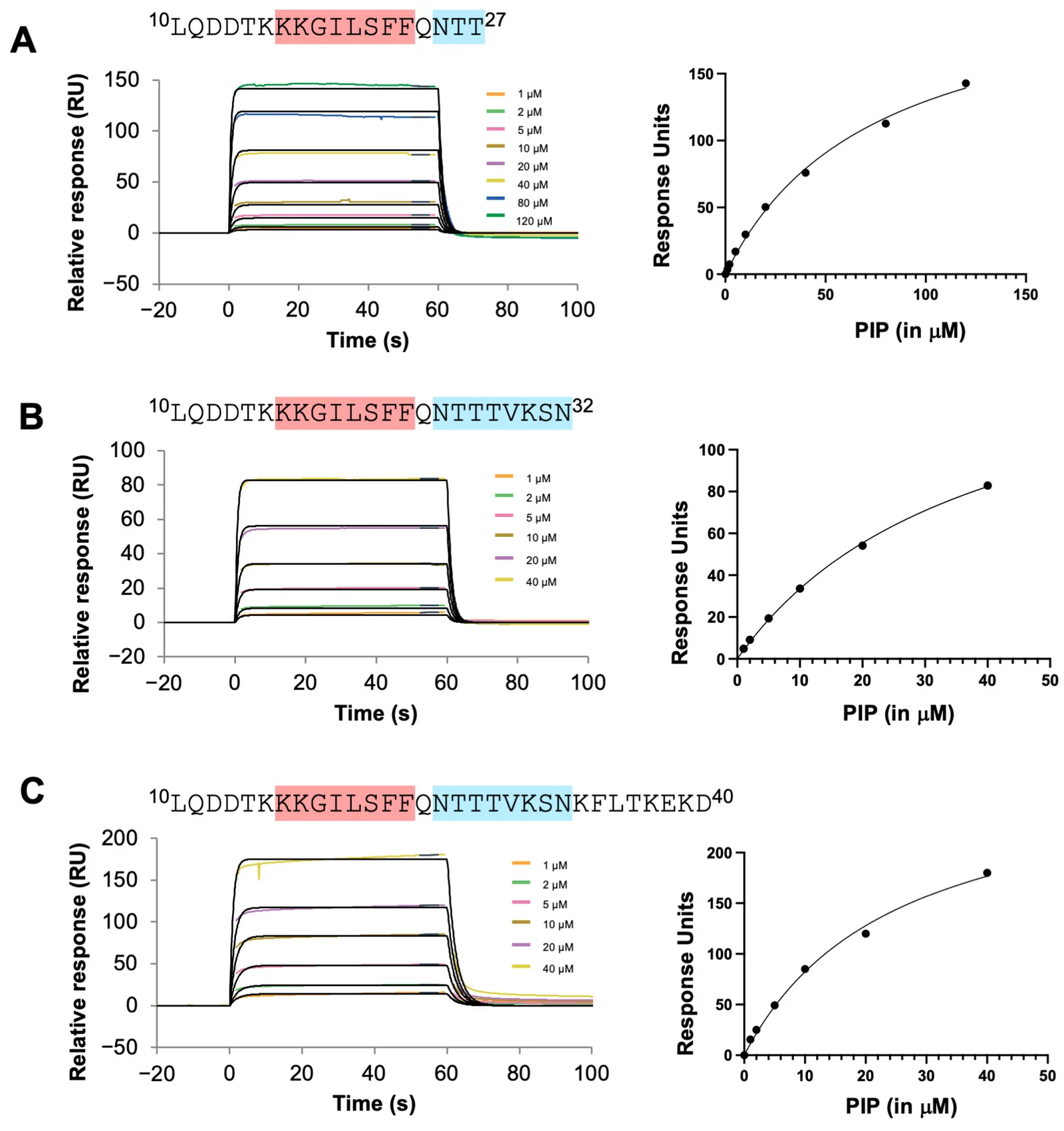
SPR binding studies of various-length CAF-1 PIP1 peptides with PCNA. Results for the PIP1 (10–27) peptide (**A**), the PIP1 (10–32) peptide, (**B**) or the PIP1 (10–40) peptide (**C**) binding to immobilized PCNA. Left: the PIP1 peptides were injected at increasing concentrations onto PCNA captured on the chip. The signal from injection of buffer only (no PIP1 peptide) was subtracted from all data. Resulting curves were fit to a one-step binding equation (BIAevaluation software program; black lines). Right: responses at equilibrium for each concentration of PIP1 peptide were plotted and fit to a non-linear regression to obtain the steady state affinity of the interaction. The CAF-1 peptide sequences used are indicated in each panel, where amino acids in the PIP1 motif are highlighted in pink, and the extended residues predicted to bind to PCNA via AlphaFold are highlighted in blue.

Compared to the peptide used in the initial SPR experiments (Figure 3), approximately a two-fold increase in affinity for PCNA was observed when only one additional residue was added to the C-terminus (residues 10–27) (Figure 6 and Table 2). Importantly, the peptide used in Figure 3 contained a carboxyl group at the C-terminus, so it is possible this negative charge interfered with a more robust interaction between PCNA and the extended residues. It is possible the addition of the two N-terminal amino acids, LQ, contributed to the increased affinity; however, the AlphaFold predicted structure of the PIP1 motif of CAF-1 bound to PCNA did not predict any interactions between the LQ of this sequence and PCNA. The affinity increased by approximately four-fold when an additional six amino acids were added to the C-terminus of the original Cac1 peptide (residues 10–32). However, no further increase in affinity was observed when an additional 14 residues were added to the C-terminus of the PIP1 motif (residues 10–40), suggesting that the peptide containing amino acids 10–32 of Cac1 is sufficient for the interaction with PCNA.

The kinetics for each extended CAF-1 peptide binding to PCNA fit best to a 1:1 binding model. As with the peptide containing only the PIP1 sequence, the association and dissociation rate constants were near the lower and upper detection limits of the instrument, respectively (Table 2). However, the quality of the fit for most experiments resulted in χ^2^ values which were less than 0.08% of the Rmax, supporting the likelihood that the kinetic values obtained are close to the true parameters. Importantly, the dissociation constants for the kinetics and the steady state analyses were also in close agreement with each other for each experiment (71 μM, 36 μM, and 30 μM for the PIP1 (10–27), PIP1 (10–32), and PIP1 (10–40) peptides, respectively).

Overall, results from these binding studies suggest that eight residues C-terminal to the PIP1 motif of CAF-1 contribute to and are sufficient for an interaction with PCNA. The presence of these residues increases the affinity of the PIP1 for PCNA and may also result in a modest, approximately two-fold faster association with PCNA compared to the PIP1 motif alone. Therefore, we propose this motif represents a novel, extended PIP motif in CAF-1. For clarity, we will refer to the PIP1 sequence alone as “PIP1” and the extended PIP1 motif (containing the additional predicted β-region residues) as “PIP1β” throughout the remainder of this manuscript.

### 3.5. The Extended PIP1 of CAF-1 Mediates Multivalent Binding to PCNA

**Figure 7:**
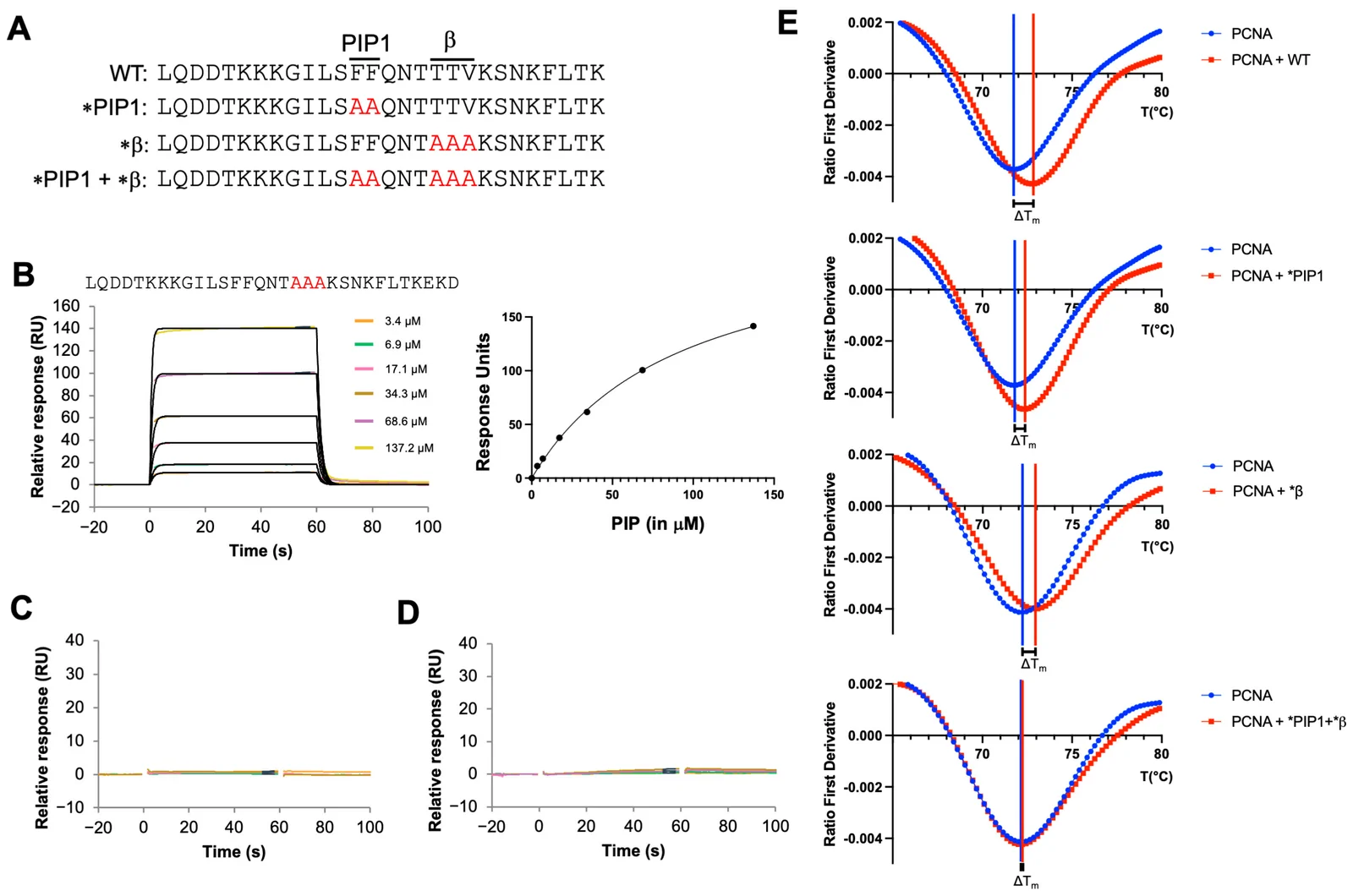
The extended PIP1β of CAF-1 is required for the full PIP–PCNA interaction. (**A**) Wildtype and mutant PIP1 peptides used for SPR and nanoDSF experiments. Mutated residues in the PIP1 or β-region are highlighted in red. (**B**) SPR results for the *β mutant PIP1 peptide binding to immobilized PCNA. The peptide sequence used for experiments in panel B, not listed in panel A, is also shown. (**C**) SPR results for the *PIP1 mutant peptide binding to immobilized PCNA. No binding was observed. (**D**) SPR results for the *PIP1 + *β mutant peptide binding to immobilized PCNA. No binding was observed. (**E**) First derivative plots of the thermal denaturation of PCNA alone (blue) or in the presence of wildtype or mutant PIP1 peptide (red) using nanoDSF. The difference between the melting temperature (T_m_) of PCNA with and without peptide is indicated by the line through the inflection point of each curve.

Based on the AlphaFold predicted structure, three amino acids—T27, T28, and V29—are likely important for formation of the β-sheet with the IDCL of PCNA (Figure 5C). We aimed to determine the contribution of each region of the PIP1β motif to the interaction with PCNA. To do this, we performed protein–protein binding studies with PCNA and CAF-1 peptides containing mutations in the PIP1 motif (containing the F22A/F23A double substitution), the β-region (containing a T27A/T28A/V29A triple substitution), or both regions simultaneously (containing F22A/F23A/T27A/T28A/V29A substitutions) (Figure 7A).

We first performed SPR using these constructs. However, only the peptide with substitutions in the β-region (*β) provided measurable affinity and kinetic parameters for binding PCNA, with K_d_ = 94 ± 6 μM, k_1_ = 1.1 × 10^4^ ± 1.4 × 10^3^ M^−1^s^−1^, and k_−1_ = 0.70 ± 0.02 s^−1^ (Figure 7B–D), an affinity intermediate to what was observed for the two PIP1 motif peptides containing a partial β-region (the PIP1 (12–26) and PIP1 (10–27) peptides) and association kinetics similar to the PIP1 (10–27) peptide. This suggests that the β-sheet–IDCL interaction alone is too weak for SPR to reliably measure binding kinetics or affinity. Thus, we switched to nano differential scanning fluorimetry (nanoDSF) to qualitatively measure the contribution of each interaction relative to each other (Figure 7E). Wildtype or mutant PIP1 peptide was incubated with PCNA, and the melting temperature (T_m_) of the complex was measured compared to PCNA alone (ΔT_m_). Larger increases in ΔT_m_ indicate greater stabilization of the PCNA–peptide complex and are therefore consistent with higher peptide affinity for PCNA.

As expected, the wildtype CAF-1 peptide bound PCNA with the highest affinity (Figure 7E and Table 3). The peptide containing only a functional PIP1 motif (*β) bound with the second-highest affinity, followed by the peptide containing only a functional β-region interaction (*PIP1). This suggests that the PIP sequence portion of the PIP1β motif of CAF-1 binds PCNA tighter than the β-region does. Finally, the CAF-1 peptide containing mutations within both the PIP1 and the β-region (*PIP1 + *β) shows almost no change in melting temperature compared to PCNA alone, suggesting that both regions of CAF-1 are required for the full, extensive interaction with PCNA.

**Figure 8:**
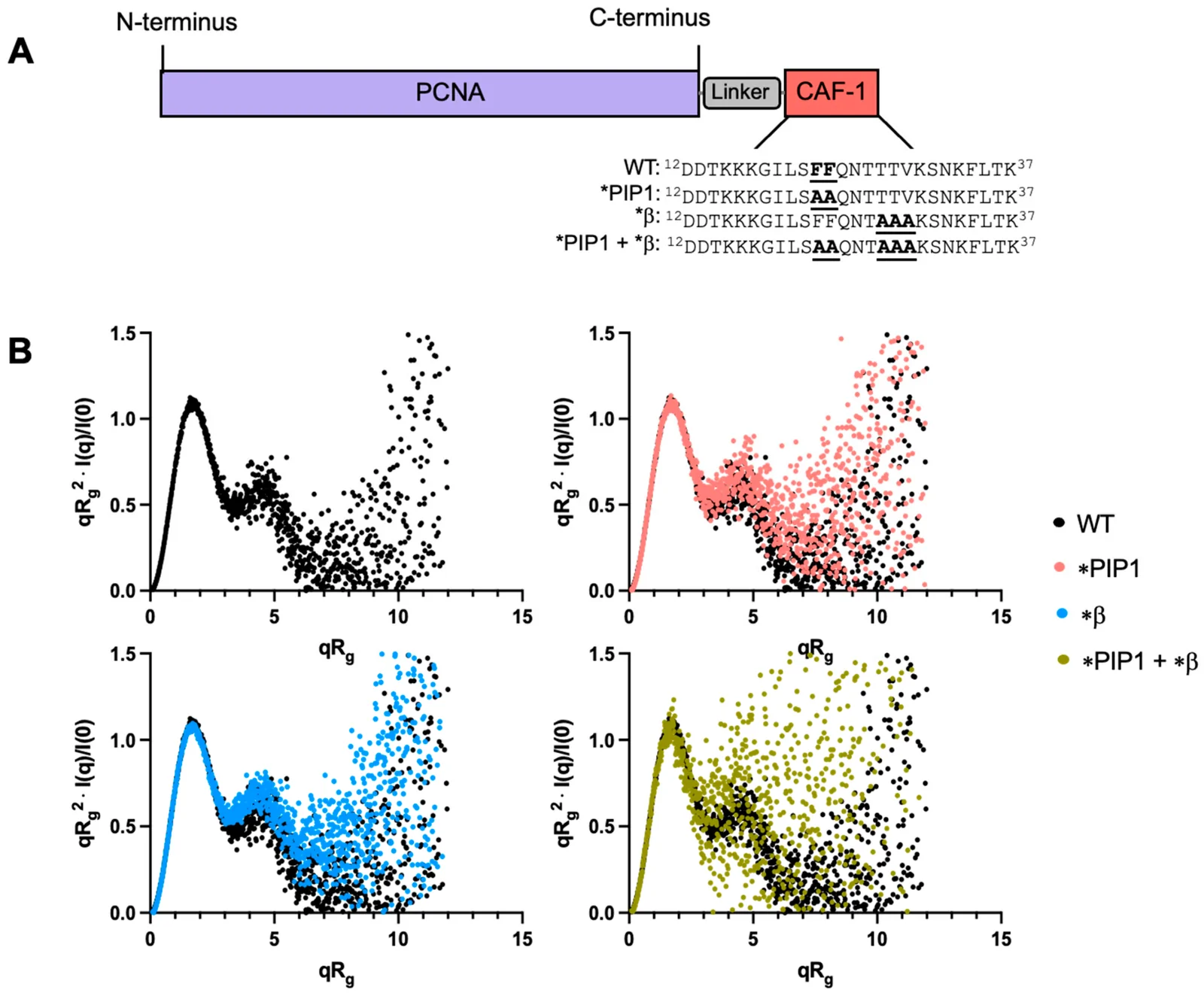
SAXS analysis of mutant CAF-1 PIP1 motifs bound to PCNA. (**A**) Linear representation of the PCNA–CAF-1 fusion proteins generated for SAXS experiments. The wildtype and PIP1 mutant sequences are indicated, with the mutated residues bolded and underlined. (**B**) Dimensionless Kratky plots for each of the mutant PCNA–CAF-1 fusion proteins overlaid with the wildtype protein.

To determine whether the overall conformation of the PIP1β–PCNA complex changes in the presence and absence of each interacting region, we carried out SAXS using similar PIP–PCNA fusion proteins, as shown in Figure 4. For this, we generated fusion proteins containing the single β-region and double PIP1 + β-region substitutions in CAF-1 (Figure 8A). The top panels in Figure 8B are the same data presented in Figure 4B,C, for easier comparison. Dimensionless Kratky analysis showed that a single mutation of either the PIP1 or the β-region alone (*PIP1 or *β) did not appreciably alter this conformational landscape, as these plots superimpose well onto the plot for wildtype protein. In contrast, simultaneous mutation of both the PIP1 and the β-region (*PIP1 + *β) shows subtle differences in the intermediate and high qRg ranges compared to the wildtype complex (bottom right panel in Figure 8B). Notably, the secondary peak is disrupted, and the scattering intensity has a very broad plateau at higher values of qRg, indicating increased conformational flexibility and disorder. This suggests that simultaneous loss of both the PIP1 and the β-region interactions reduces the degree to which CAF-1 engages PCNA in this construct, where either no or fewer interactions are occurring between the two proteins. It is important to note that the structure of PCNA does not change substantially upon binding PIP motifs [5,11,31], so a drastic change was not expected. Together, these results demonstrate that the PIP1β motif of CAF-1 mediates multivalent binding to PCNA, where the PIP1 and extended β-region residues combined have a greater affinity for PCNA than either motif alone.

### 3.6. The PIP1 and PIP2 Motifs Are Both Required for CAF-1 Function In Vivo

**Figure 9:**
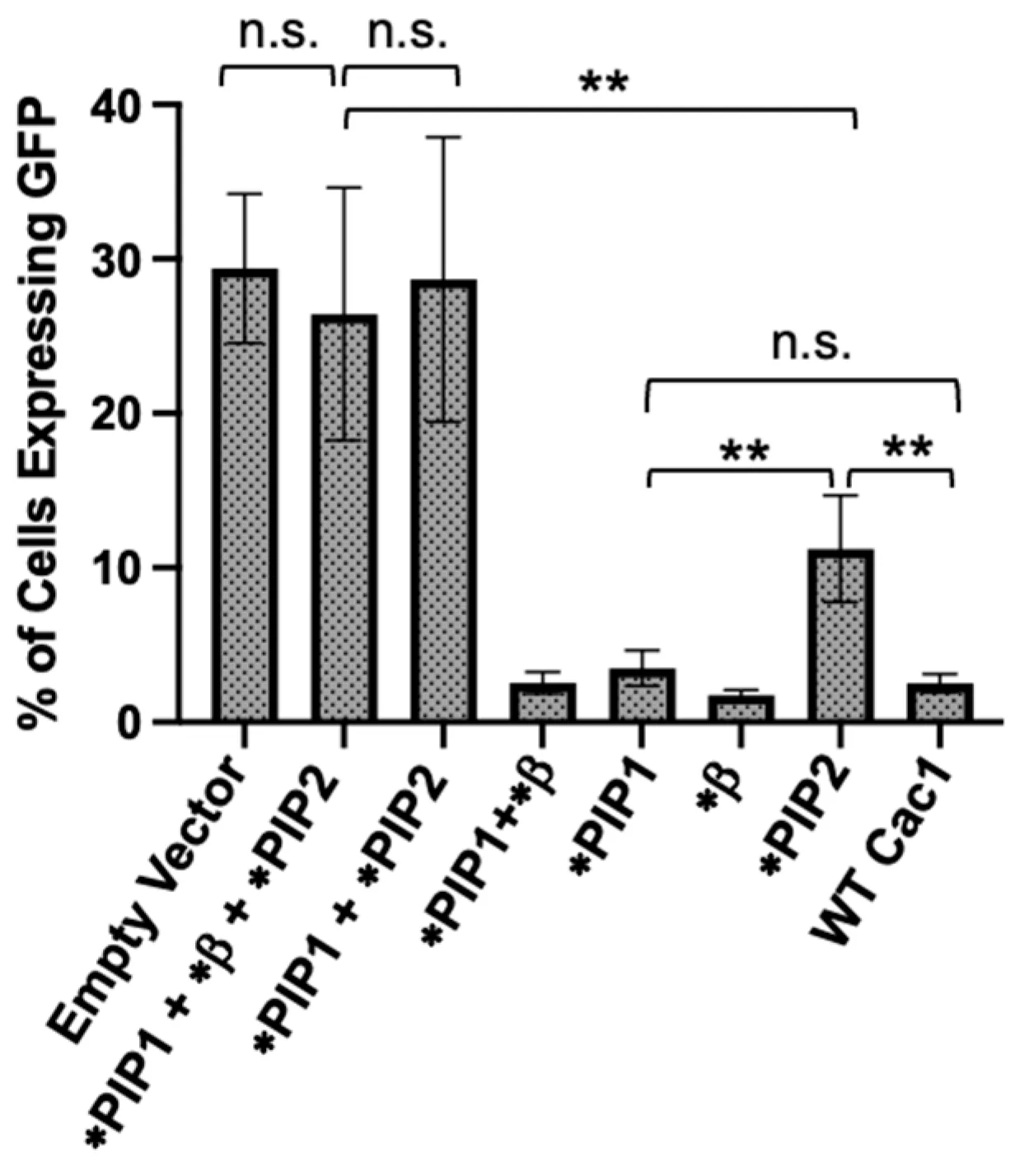
Effects of mutant CAF-1 proteins on gene silencing. Silencing of GFP by wildtype Cac1 (WT Cac1) or mutant Cac1 proteins (denoted by asterisks) was measured by flow cytometry. GFP-expressing yeast cells containing the pRS313 vector only (empty vector) were used as a control. Loss of CAF-1 function is shown as increased expression of GFP (compare empty vector to WT Cac1). Data are presented as mean ± standard deviation of n ≥ 3 independent experiments. Significance was determined using an unpaired *t*-test. ** *p* < 0.05, n.s., not significant. Note that all samples with a percentage of cells expressing GFP above 20% (the first three columns) were not significantly different from each other, and all samples with a percentage of cells expressing GFP below 10% (the last five columns, excluding the *PIP2 sample) were also not significantly different from each other. These comparisons were not included in the figure to avoid visual clutter and maintain readability.

Finally, we investigated the importance of the PIP1 motif in the function of CAF-1 in gene silencing in vivo. We used a silencing assay in *Saccharomyces cerevisiae* in which a GFP reporter was integrated at the *HMR* locus in cells lacking endogenous Cac1 (*cac1Δ*). Loss of Cac1 disrupts CAF-1-dependent silencing, resulting in GFP expression that can be quantified by flow cytometry. Expression of wildtype Cac1 from a plasmid restored GFP silencing, confirming functional complementation (Figure 9, compare WT Cac1 and empty vector).

To determine the contributions of the PIP1 motif, its flanking β-region, and the previously characterized PIP2 motif, we generated Cac1 variants containing substitutions in (1) the PIP1 motif alone (*PIP1; FF22/23AA); (2) the β-region alone (*β; TTV27–29AAA); (3) both the PIP1 motif and the β-region (*PIP1 + *β; FF22/23AA, TTV27–29AAA); (4) the PIP2 motif alone (*PIP2; FF233/234AA); (5) both PIP1 and PIP2 (*PIP1 + *PIP2; FF22/23AA, FF233/234AA); or (6) PIP1, the β-region, and PIP2 (*PIP1 + *β + *PIP2; FF22/23AA, TTV27–29AAA, FF233/234AA). Each construct was expressed in *cac1Δ* cells, and GFP fluorescence was quantified as a measure of silencing.

Mutation of any individual element—PIP1, the β-region, or PIP2—did not substantially impair CAF-1-dependent silencing. Consistent with our previous observations [11], the PIP2 mutant exhibited a modest reduction in silencing relative to wildtype Cac1. Similarly, simultaneous mutation of PIP1 and the β-region (*PIP1 + *β) did not impair silencing, indicating that disruption of PIP1-mediated PCNA binding alone is insufficient to compromise CAF-1 function in vivo. In contrast, simultaneous mutation of PIP1 and PIP2, or of PIP1, the β-region, and PIP2, resulted in a severe silencing defect comparable to that observed in the absence of CAF-1. This suggests that the PIP1 or PIP1β and the PIP2 motifs together are essential for CAF-1 function in vivo, while the individual PIP motifs are not required for gene silencing at the *HMR* locus.

## 4. Discussion

In this study, we identified a second PIP motif in yeast CAF-1. This motif exists near the N-terminus of the Cac1 subunit in an intrinsically disordered region. We designated this new PIP motif as PIP1, as the previously known PIP motif (designated as “PIP2”) is located C-terminal to the PIP1 motif. CAF-1 represents one of only a few known yeast proteins that contain multiple PIP motifs, most notably DNA polymerase η (pol η) [22]. As with pol η, the two PIP motifs in CAF-1 bind PCNA with substantially different affinities and kinetics, where the motifs with weaker affinity (the PIP1 of CAF-1 and the PIP2 of pol η, respectively) also bind PCNA with faster kinetics. This suggests that the two motifs in each protein carry out differing roles in pol η and CAF-1 during DNA replication and nucleosome assembly, respectively. A similar division of labor between multiple PIP motifs has recently been proposed, where a single internal PIP motif makes the critical PCNA contact, while PIP motifs located in long disordered regions may function as flexible tethers that stabilize the interactions of these proteins to PCNA [32]. Our finding that the PIP2 motif of CAF-1 has higher affinity for PCNA than the extended PIP1β motif is consistent with this proposed functional hierarchy, in which one PIP motif serves as the primary anchor while the other supports a more dynamic or auxiliary role.

We also determined that the novel PIP1 motif of CAF-1 is not a standard PIP motif, but is instead an extended PIP motif that makes substantial interactions with the IDCL of PCNA. AlphaFold predicts a β-sheet is formed between these additional residues of the PIP1 motif and PCNA, and we therefore designated the full-length PIP1 motif as the PIP1β motif. We determined that this motif binds PCNA with an affinity of approximately 35 μM. There are only a few other PCNA-binding proteins that interact substantially with the IDCL of PCNA to form β-sheets, most notably p21 and FEN1. The extended PIP motifs of p21 and FEN1 bind PCNA in the sub-micromolar (0.08–0.3 μM) and mid-micromolar range (10–60 μM), respectively [27]. In the case of p21, it has been suggested that these extensive interactions are responsible for the high-affinity interaction with PCNA relative to other PIP motifs [4,5]. Although the additional interactions increase the affinity of these proteins to PCNA compared to the PIP motif alone, extended PIP motifs do not always appear to increase affinity to a range higher than other canonical, non-extended PIP motifs. Instead, this suggests that the extended motifs may be important for PCNA selectivity of binding partners at the replication fork during DNA metabolic processes. Furthermore, this growing structural diversity among PIP motifs is consistent with the broader view that PIP and related PCNA-binding motifs should not necessarily be viewed as rigid, sequence-defined interaction modules. Rather, they can exhibit sequence context outside the canonical motif contributing to target recognition and binding affinity [33].

Two PIP motifs exist in human CAF-1, hPIP1 and hPIP2. These two motifs exist in locations within the p150 subunit that are similar to the locations of the yeast PIP1 and PIP2 in Cac1 (Figure 2). Beyond this, there are few similarities between the yeast PIP1 and hPIP1 motifs and the yeast PIP2 and hPIP2 motifs. The hPIP1 sequence (QARLFF) is more similar to the yeast PIP2 sequence (QSRIGNFF), whereas the hPIP2 sequence (KAEITRFF) is closer in sequence to the yeast PIP1 (KKGILSFF). We determined that the yeast PIP1 motif binds PCNA with approximately 10-fold lower affinity than the yeast PIP2 motif, despite the presence of an extended yeast PIP1β sequence. We also show that the yeast PIP1β motif binds PCNA via a transient, one-step binding mechanism, compared to the slower, two-step yeast PIP2–PCNA binding mechanism. In contrast, hPIP1 does not appear to interact with the IDCL of PCNA [34], but it has a higher affinity for PCNA than the hPIP2 motif. No precise K_d_ values or kinetics have been determined for either hPIP1 or hPIP2, but studies show that the hPIP2–PCNA interaction is transient [16]. Together, this demonstrates that the yeast PIP1β−PCNA interaction is more similar to the hPIP2–PCNA interaction, and that the yeast PIP2–PCNA is more similar to the hPIP1–PCNA interaction. In terms of function, hPIP1 has been shown to be dispensable for nucleosome assembly in vivo, while hPIP2 is critical for nucleosome assembly and targeting of CAF-1 to the replication fork [10,16]. In contrast, the PIP1β and PIP2 PCNA-binding elements make partially redundant contributions to CAF-1-dependent gene silencing in vivo: disruption of either element alone is tolerated, whereas simultaneous disruption of both severely compromises silencing at the *HMR* locus. It should be noted that our experiments establish the functional importance and redundancy of these PIP motifs in vivo, but they do not specifically describe relative contributions during post-replicative chromatin assembly. Overall, the two PIP motifs in CAF-1 have differing functions from each other in both humans and yeast. Moreover, data suggest that the PCNA-binding activity of hPIP1 is more similar to, but still distinct from, that of yeast PIP2, and the same is true for hPIP2 and yeast PIP1β. However, the PIP2 motif appears to be more significant for the function and/or recruitment of CAF-1 in nucleosome assembly in both organisms. More studies are needed to both understand these differences and parse out the roles each motif plays in regulating the activity of CAF-1.

**Figure 10:**
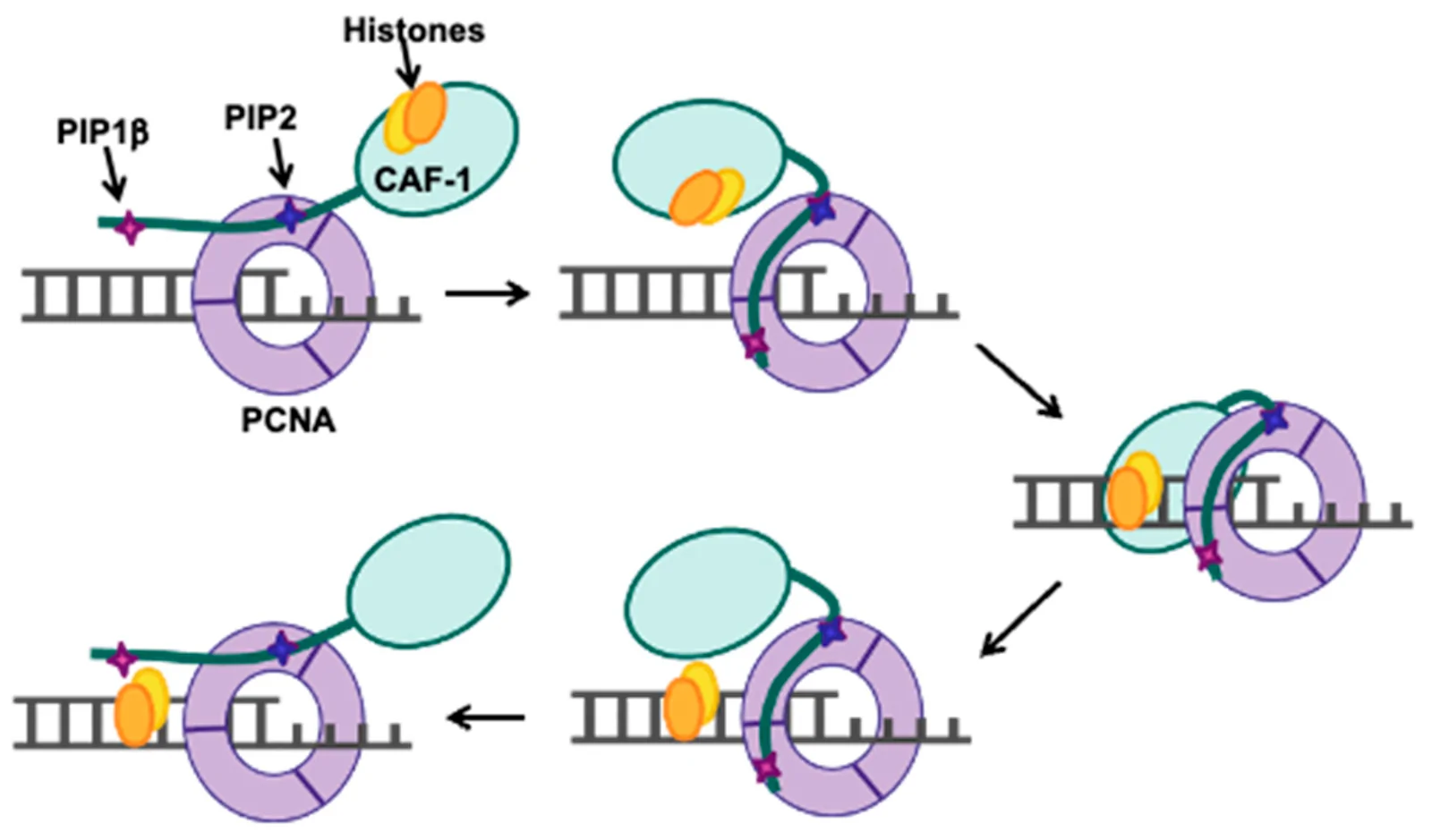
Proposed mechanism of histone deposition by yeast CAF-1 using two PIP motifs. CAF-1 (teal) bound to histone proteins (orange/yellow) is recruited to PCNA (purple) on DNA via an interaction between the PIP2 motif of CAF-1 (blue) and one subunit of PCNA. A conformational change occurs in the complex where the PIP1β (magenta) transiently interacts with a different subunit of PCNA, and CAF-1 deposits histones on the DNA behind PCNA. The PIP2–PCNA interaction may remain or be released during this step. Finally, the PIP1β–PCNA interaction is released, and the PIP2 motif of CAF-1 can either remain bound to PCNA or dissociate before the next round of nucleosome assembly. This mechanism may also be utilized with any two PIP motifs in a complex containing two CAF-1 heterotrimers.

Based on our in vivo and kinetics studies of the PIP1β–PCNA and PIP2–PCNA interactions, we propose that the PIP2 motif performs the predominant role in recruiting yeast CAF-1 to replication forks. We propose that PIP2 binding to PCNA increases the local concentration of PIP1β, enabling it to bind a separate subunit of PCNA and triggering a conformational change across the CAF-1 complex that drives histone deposition onto nascent DNA behind the replication fork (Figure 10). The extended β-sheet formed between the PIP1β and PCNA might be necessary for histone deposition, as additional contacts with the IDCL could help stabilize the transition of CAF-1 to the back of PCNA when nucleosome formation is required. Since both PIP motifs are located within disordered regions of the protein, this would allow flexibility for CAF-1 to transition from the front to the back of PCNA. Alternatively, binding of PIP1β to PCNA may prevent other proteins from being recruited to the DNA. After histone deposition, CAF-1 may be released, or PIP2 may hold the PCNA–CAF-1 complex together for the next round of nucleosome assembly as the replication fork proceeds. This process would not interfere with DNA replication, as at least one subunit of PCNA would still be available to bind DNA polymerases.

It is interesting that our in vivo results show that one PIP motif can rescue the activity of CAF-1 in the absence of the other PIP motif (Figure 9). CAF-1 is believed to function as a dimer (requiring two heterotrimers) at the replication fork [35,36]. It is therefore possible that a CAF-1 complex containing a non-functional PIP motif can still be recruited to PCNA as long as two functional PIP motifs are present in the entire complex. Here, the handoff of CAF-1 from the front to the back surface of PCNA via binding two different subunits of the PCNA trimer is still feasible.

Previous studies have identified and determined the structure of a mutant form of PCNA that does not bind CAF-1 and is defective in gene silencing, L126A/I128A [8,12]. This mutation does not appear to affect other cellular processes, suggesting that the defect in interaction is specific to CAF-1. L126 and I128 both exist in the IDCL of PCNA. Because both the PIP1 and PIP2 motifs of CAF-1 interact more extensively with the IDCL than canonical PIP motifs, the L126A/I128A mutation likely hinders both motifs from binding. This would explain why the L126A/I128A mutant PCNA protein is still able to bind other PIP-containing proteins but not CAF-1.

## 5. Conclusions

It is unclear how PCNA selectively recruits CAF-1 to replication forks, especially given the large number of PIP-containing proteins competing for access to PCNA. Our findings suggest that the presence of drastically different PIP motifs—including differences in sequence, length, binding kinetics, affinities, and secondary structure formation—may aid PCNA in the temporal and spatial selection of CAF-1 during replication-coupled nucleosome assembly. More broadly, it is likely that these same principles govern other cellular processes that require temporal and spatial discrimination by PCNA.

## Figures and Tables

**Table 1:** Binding kinetics and affinities of the PIP1 and PIP2 motifs for PCNA.

CAF-1 Peptide	k_1_ (M^−1^s^−1^)	k_−1_ (s^−1^)	k_2_ (M^−1^s^−1^)	k_−2_ (s^−1^)	K_d_ (μM)
PIP1	9.4 × 10^3^	1.1	N/A	N/A	120 ± 10
PIP2 ^a^	1.4 × 10^5^	7.4 × 10^−1^	6.2 × 10^−4^	1.7 × 10^−3^	4

N/A = not applicable. ^a^ Values previously determined [11].

**Table 2:** Binding kinetics and affinities of PIP1 peptides for PCNA.

CAF-1 Peptide	k_1_ (M^−1^s^−1^)	k_−1_ (s^−1^)	K_d_ (μM)
PIP1 (10–27)	1.0 × 10^4^ ± 1.6 × 10^3^	0.60 ± 0.02	69 ± 2
PIP1 (10–32)	2.2 × 10^4^ ± 1.9 × 10^3^	0.97 ± 0.07	34 ± 2
PIP1 (10–40)	1.9 × 10^4^ ± 8.6 × 10^2^	0.54 ± 0.06	32 ± 2

**Table 3:** Melting temperatures of PCNA bound to PIP1 peptides.

CAF-1 Peptide	ΔT_m_/°C
WT	1.12 ± 0.17
*PIP1	0.54 ± 0.18
*β	0.89 ± 0.14
*PIP1 + *β	0.13 ± 0.15

## Data Availability

All data generated for this study are included in this article.

## Supplementary Materials

The following supporting information can be downloaded at: https://www.mdpi.com/article/10.3390/biom16091274/s1, Figure S1: Characterization of synthesized peptides; Table S1: Sequences and sources of peptides used in binding studies; Table S2: Sequences of PCNA and PCNA-PIP1 fusion proteins.

## Abbreviations

The following abbreviations are used in this manuscript:

PCNA	Proliferating cell nuclear antigen
CAF-1	Chromatin assembly factor 1
PIP	PCNA-interacting protein
IDCL	Interdomain connecting loop
IDR	Intrinsically disordered region
SPR	Surface plasmon resonance
SAXS	Small-angle X-ray scattering
DSF	Differential scanning fluorimetry
GFP	Green fluorescent protein
HCTU	O-(1H-6-chlorobenzotriazole-1-yl)-1,1,3,3- tetramethyluronium hexafluorophosphate
DMF	N, N-dimethylformamide
TFA	Trifluoroacetic acid
HPLC	High-performance liquid chromatography
ACN	Acetonitrile
SEC	Size exclusion chromatography
MALS	Multiangle light scattering
DLS	Dynamic light scattering
RI	Refractive index
YPD	Yeast peptone dextrose
FCS	Flow cytometry standard
