# Supplementary material for: Identification of a Second PIP Motif Reveals New Insights into PCNA Recognition by Yeast CAF-1
Source: Biomolecules. 2026 Sep 3;16(9):1274. doi: 10.3390/biom16091274 (PMC13604413; doi:10.3390/biom16091274)
Supplement: Supplementary file 1 [file biomolecules-16-01274-s001.zip › biomolecules-4478841-supplementary.pdf]

Supplementary Figures

Figure S1. Characterization of synthesized peptides. For each peptide, the top panel shows evaluation of peptide purity by analytical-scale reverse-phase high-performance liquid chromatography (HPLC), as assessed by UV absorbance at 220 nm. The HPLC conditions for each chromatographic run were: solvent A = water + 0.1% trifluoroacetic acid, solvent B = acetonitrile + 0.1% trifluoroacetic acid, gradient = 5-55% B over 30 or 60 min.

The bottom panel shows confirmation of peptide identity by matrix-assisted laser desorption ionization mass spectrometry using the monoisotopic $[M+H]^+$ peak in each spectrum.

(A) WT PIP1 peptide (10 – 27), calculated monoisotopic mass $[M+H]^+$ = 2269.197 m/z.

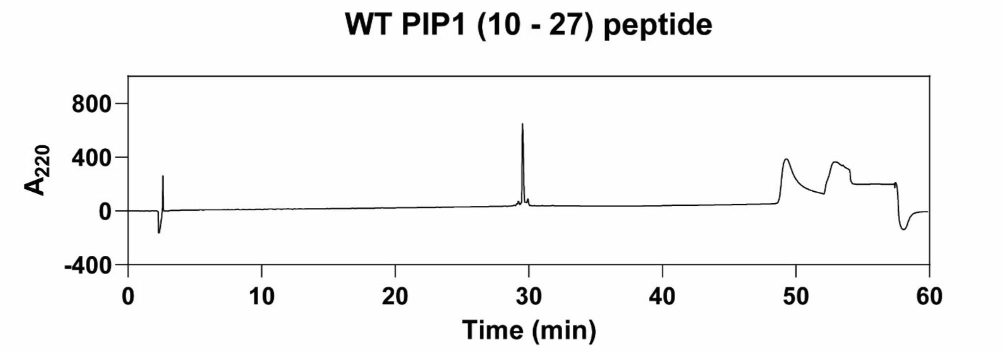

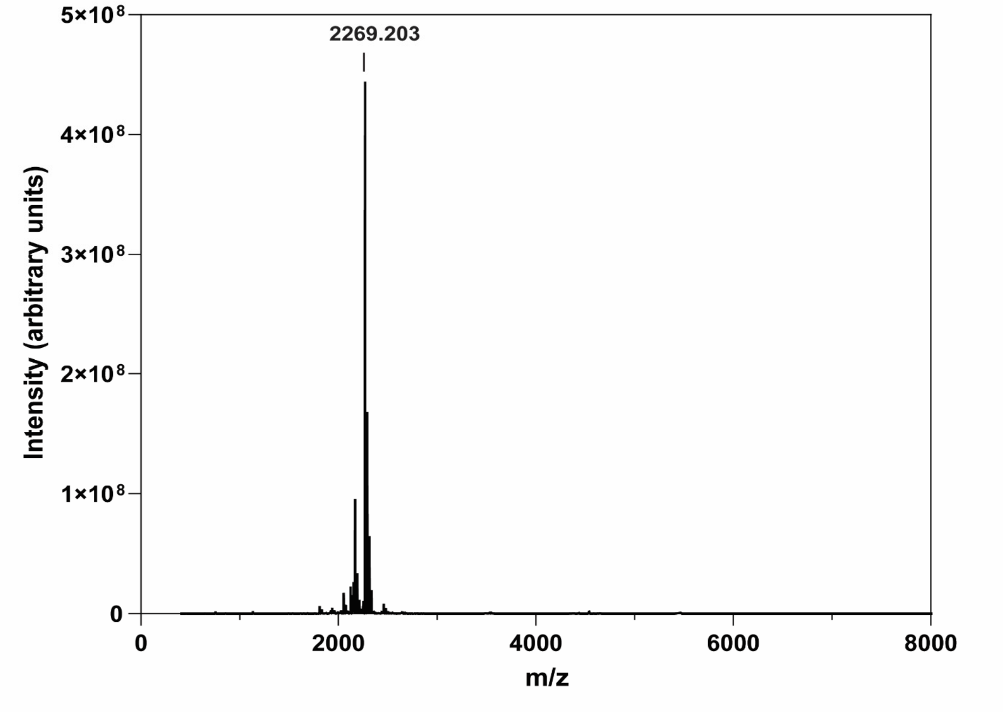

(B) WT PIP1 peptide (10 – 32), calculated monoisotopic mass $[M+H]^+ = 2798.484$ m/z.

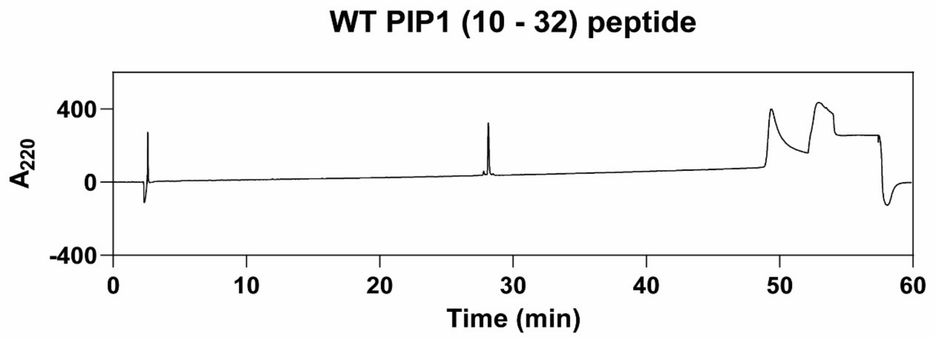

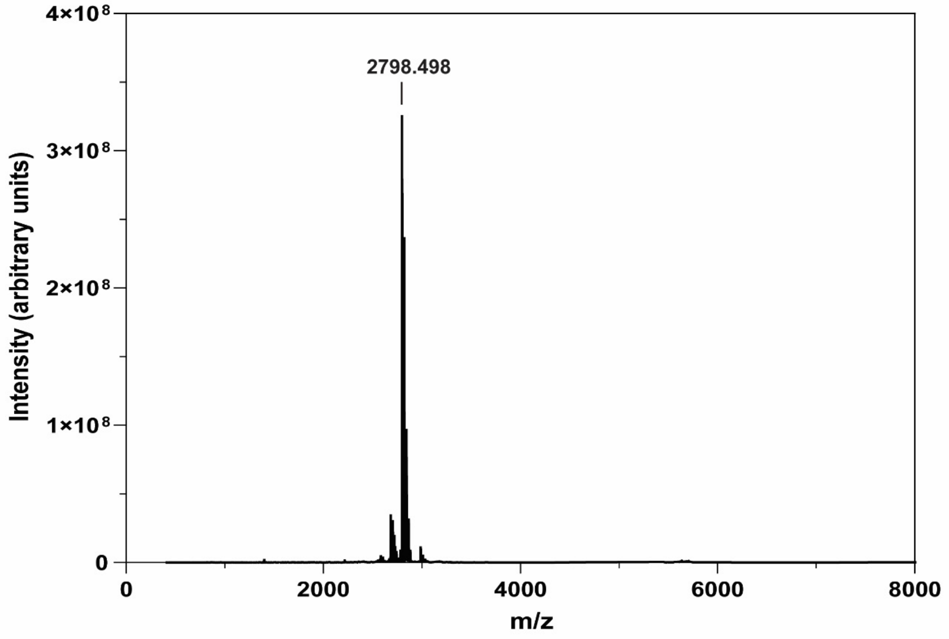

(C) WT PIP1 peptide (10 – 40), calculated monoisotopic mass $[M+H]^+$ = 3788.038 m/z.

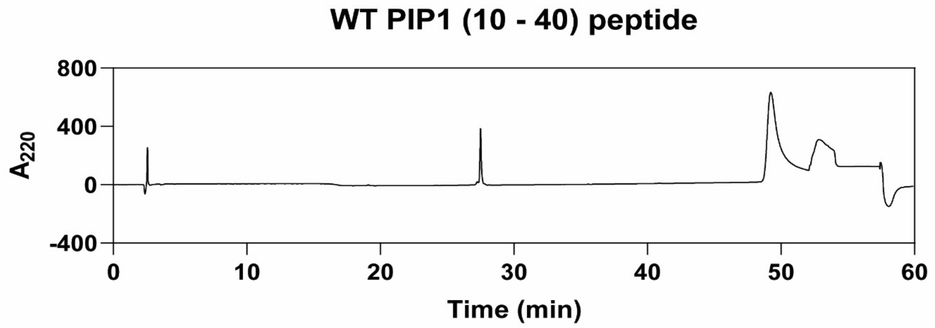

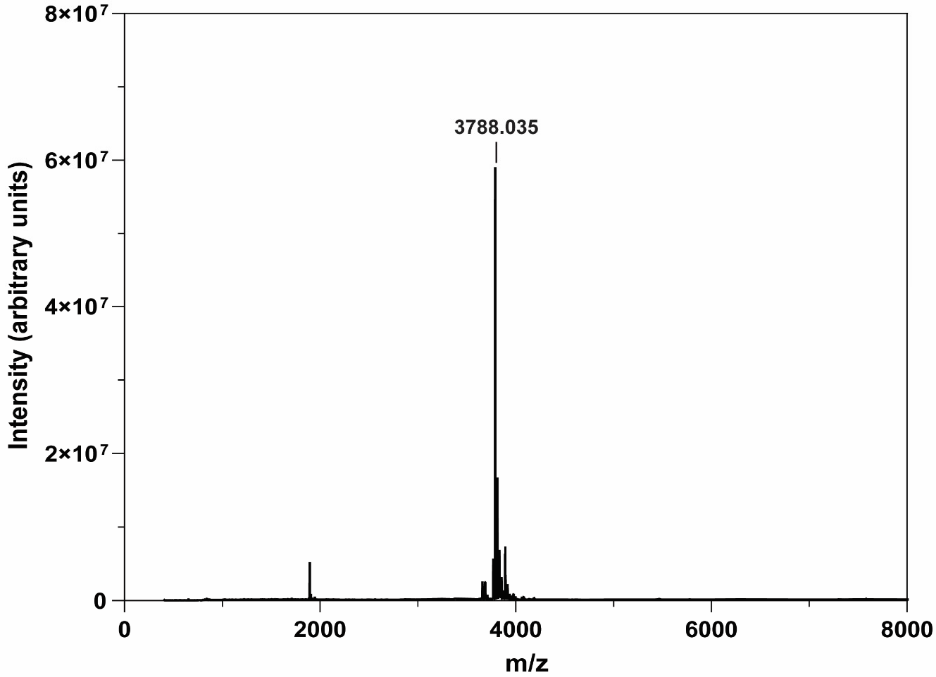

(D) *β mutant PIP1 (10 - 40) peptide, calculated monoisotopic mass $[M+H]^+$ = 3513.906 m/z.

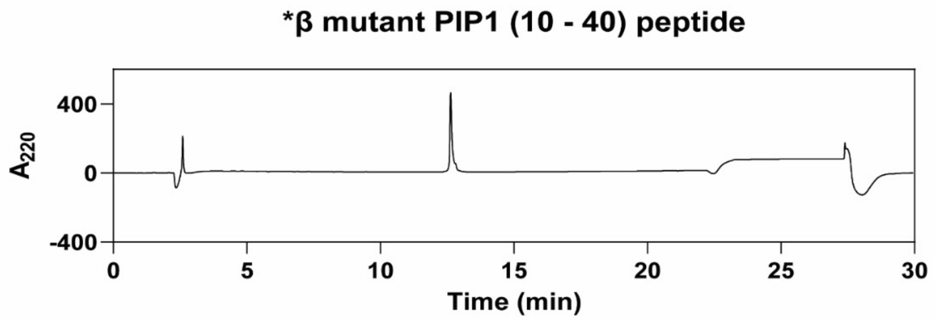

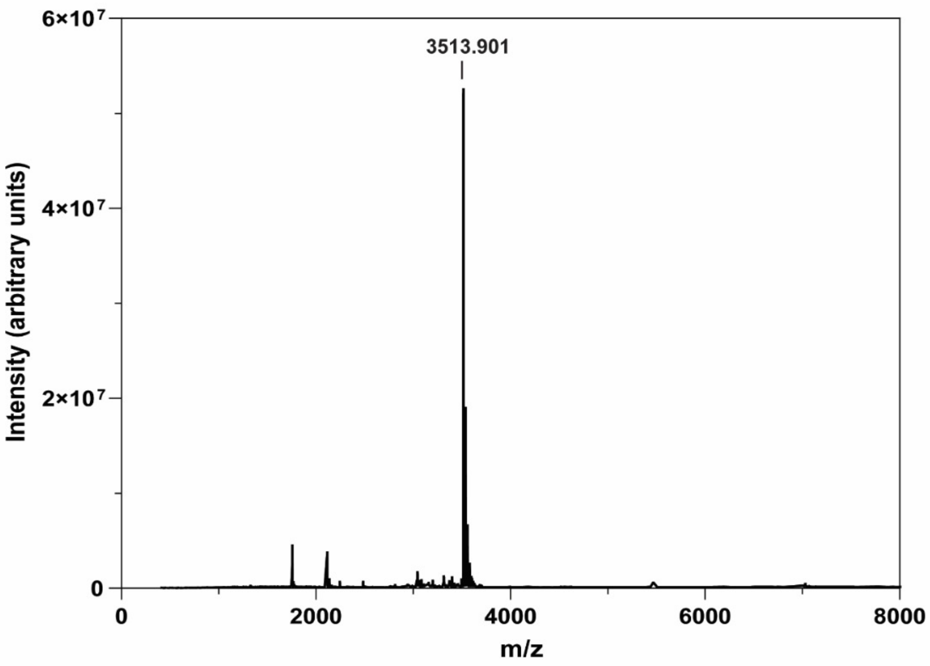

Table S1. Sequences and sources of peptides used in binding studies.

Figure	Peptide sequence	Source of peptide
3	WDDTKKKGILSFFQNT-OH	GenScript
6A	WLQDDTKKKGILSFFQNTT-NH_2	Checco Lab
6B	WLQDDTKKKGILSFFQNTTTVKSN-NH_2	Checco Lab
6C	WLQDDTKKKGILSFFQNTTTVKSNKFLTKEKD-NH_2	Checco Lab
7B	LQDDTKKKGILSFFQNTAAAKSNKFLTKEKD-NH_2	Checco Lab
7C	LQDDTKKKGILSAAQNTTTVKSNK-OH	GenScript
7D	LQDDTKKKGILSAAQNTAAAKSNK-OH	GenScript
7E	LQDDTKKKGILSFFQNTTTVKSNK-OH<br>LQDDTKKKGILSFFQNTAAAKSNK-OH<br>LQDDTKKKGILSAAQNTTTVKSNK-OH<br>LQDDTKKKGILSAAQNTAAAKSNK-OH	GenScript

Note: peptides containing tryptophan were excluded from nanoDSF experiments because tryptophan fluorescence can interfere with the intrinsic fluorescence signal used to monitor PCNA thermal unfolding.
NH_2 = amide
OH = carboxylic acid

Table S2. Sequences of PCNA and PCNA-PIP1 fusion proteins.

Protein	Sequence
His_{12}-PCNA	(MHHHHHHHHHHHH)MLEAKFEEASLFKRIIDGFKDCVQLVNFQCKEDGIIA<br>QAVDDSRVLLVSLEIGVEAFQEYRCDHPVTLGMDLTSLSKILRCGNNTDTLT<br>LIADNTPDSIILLFEDTKKDRIAEYSLKLMDIDADFLKIEELQYDSTLSLPS<br>SEFSKIVRDLSQLSDSINIMITKETIKFVADGDIGSGSVIIKPFVDMEHPET<br>SIKLEMDQPVDLTFGAKYLLDIIKGSSLSDRVGIRLSSEAPALFQFDLKSGF<br>LQFFLAPKFNDEE
His_6-PCNA-PIP1	(MHHHHHH)MLEAKFEEASLFKRIIDGFKDCVQLVNFQCKEDGIIAQAVDDS<br>RVLLVSLEIGVEAFQEYRCDHPVTLGMDLTSLSKILRCGNNTDTLTLIADNT<br>PDSIILLFEDTKKDRIAEYSLKLMDIDADFLKIEELQYDSTLSLPSSEFSKI<br>VRDLSQLSDSINIMITKETIKFVADGDIGSGSVIIKPFVDMEHPETSIKLEM<br>DQPVDLTFGAKYLLDIIKGSSLSDRVGIRLSSEAPALFQFDLKSGFLQFFLA<br>PKFNDEEGGSGGDDTKKKGILSFFQNTTTVKSNKFLTK

Histidine tags are shown in parentheses.
